# Electrospun Resveratrol-Loaded Polyvinylpyrrolidone/Cyclodextrin Nanofibers and Their Biomedical Applications

**DOI:** 10.3390/pharmaceutics12060552

**Published:** 2020-06-13

**Authors:** Ying-Cheng Lin, Stephen Chu-Sung Hu, Pao-Hsien Huang, Tzu-Ching Lin, Feng-Lin Yen

**Affiliations:** 1 Department of Fragrance and Cosmetic Science, College of Pharmacy, Kaohsiung Medical University, Kaohsiung 807, Taiwan; a5710736@gmail.com (Y.-C.L.); cheng22701@gmail.com (T.-C.L.); 2 Graduate Institute of Toxicology, College of Medicine, National Taiwan University, Taipei 100233, Taiwan; 3 Department of Dermatology, College of Medicine, Kaohsiung Medical University, Kaohsiung 807, Taiwan; stephen@kmu.edu.tw; 4 Department of Dermatology, Kaohsiung Medical University Hospital, Kaohsiung 807, Taiwan; 5 Drug Development and Value Creation Research Center, Kaohsiung Medical University, Kaohsiung 807, Taiwan; 6 Department of Medical Research, Kaohsiung Medical University Hospital, Kaohsiung 807, Taiwan; 7 Translational Research Center, Kaohsiung Medical University Hospital, Kaohsiung Medical University, Kaohsiung 807, Taiwan; 8 Department of Dermatology, Kaohsiung Municipal Siaogang Hospital, Kaohsiung 812, Taiwan; 9 School of Pharmacy, College of Pharmacy, Kaohsiung Medical University, Kaohsiung 807, Taiwan; j6466497@gmail.com; 10 Institute of Biomedical Sciences, National Sun Yat-Sen University, Kaohsiung 804, Taiwan

**Keywords:** resveratrol, cyclodextrin, nanofiber, electrospinning, antioxidant activity

## Abstract

Resveratrol is a naturally occurring polyphenol compound which has been shown to possess antioxidant and anti-inflammatory properties. However, its pharmaceutical applications are limited by its poor water solubility. In this study, we used electrospinning technology to synthesize nanofibers of polyvinylpyrrolidone (PVP) and hydroxypropyl-β-cyclodextrin (HPBCD) loaded with resveratrol. We used X-ray diffractometry to analyze crystalline structure, Fourier transform infrared spectroscopy to determine intermolecular hydrogen bonding, antioxidant assays to measure antioxidant activity, and Franz diffusion cells to evaluate skin penetration. Our results showed that the aqueous solubility of resveratrol nanofibers was greatly improved (by more than 20,000-fold) compared to the pure compound. Analysis of physicochemical properties demonstrated that following nanofiber formation, resveratrol was converted from a crystalline to amorphous structure, and resveratrol formed new intermolecular bonds with PVP and HPBCD. Moreover, resveratrol nanofibers showed good antioxidant activity. In addition, the skin penetration ability of resveratrol in the nanofiber formulation was greater than that of pure resveratrol. Furthermore, resveratrol nanofibers suppressed particulate matter (PM)-induced expression of inflammatory proteins (COX-2 and MMP-9) in HaCaT keratinocytes. Therefore, resveratrol-loaded nanofibers can effectively improve the solubility and physicochemical properties of resveratrol, and may have potential applications as an antioxidant and anti-inflammatory formulation for topical skin application.

## 1. Introduction

**Figure 1:**
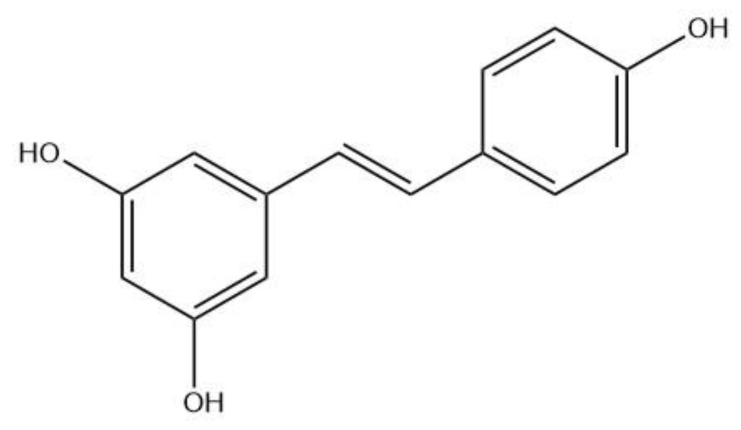
Chemical structure of resveratrol (3,5,4′-trihydroxystilbene).

Resveratrol (Res) is a naturally occurring non-flavonoid polyphenol compound and is characterized by a stilbene chemical structure (Figure 1) [1]. It is classified as a phytoalexin, an antimicrobial substance which is synthesized by plants and accumulates around sites of infection [2]. It is known to have antioxidant [3,4], anti-inflammatory [5,6], and antimicrobial properties [7,8]. Resveratrol has also been found to have protective effects on the cardiovascular system [9,10]. Moreover, it has been shown to have anticancer properties in various types of cancers [11,12,13,14]. Resveratrol is found in grapes and various other plants, and is also present in red wine. The consumption of red wine by the French people may partially explain the low incidence of coronary heart disease despite high dietary consumption of saturated fats, a phenomenon known as the French paradox [15,16,17].

A major drawback of resveratrol is that it is poorly soluble in water, and can only dissolve in organic solvents such as methanol and ethanol [18,19]. This may decrease its bioavailability and limit its potential applications in the biomedical field. Nanoparticle formulations have been used to improve the solubility of drugs, including liposomes, polymers, gold, silicon and nanofibers [20,21]. In recent years, various studies have attempted to synthesize nanoparticle formulations of resveratrol, in order to improve its water solubility, bioavailability and biological activities [22,23,24,25,26]. For example, nanoformulations of resveratrol have been used in the oncology setting to prevent and treat various types of cancer [27].

Cyclodextrins are generated from the enzymatic degradation of starch. They have a truncated cone structure with a lipophilic inner cavity and hydrophilic outer surface, leading to high aqueous solubility. They can therefore act as nanoparticle sized carriers to improve the water solubility of lipophilic drugs, and enable controlled drug release [28,29,30]. Naturally occurring cyclodextrins are categorized into α-cyclodextrin (6 glucopyranose units), β-cyclodextrin (7 glucopyranose units), and γ-cyclodextrin (8 glucopyranose units). β-cyclodextrins, such as hydroxypropyl-β-cyclodextrin (HPBCD), are most commonly used as drug nanocarriers due to their optimal cavity size and good drug loading capacity [31,32]. When a drug is included within the cavity of a cyclodextrin, an inclusion complex is generated, thus improving the solubility and stability of the drug [33].

Electrospinning technology uses electrostatic forces to generate nanofibers with very fine diameters [34,35]. There are three main components in the electrospinning system: a high-voltage generator, a syringe with micropump, and a collecting metal plate (such as aluminum foil) [36]. During the electrospinning procedure, the active compound is dissolved in a suitable solvent, and a high molecular weight polymer is added. The solution is transferred into a syringe attached to a micropump, and a high voltage is applied to induce electric charges in the solution [37]. When the electrical forces overcome the surface tension of the solution, a thin liquid jet will leave the syringe tip and travel to the collecting board (containing the opposite charge), and nanoscale fibers are generated as the solvent evaporates [38]. The nanofibers formed by electrospinning technology are characterized by high surface area to volume ratio, and applications include tissue engineering, biological sensors, wound dressings, and drug carriers [39]. In recent years, electrospinning technology has been increasingly used in the biomedical field, and is becoming a popular area of nanotechnology research [36]. In particular, state-of-the-art developments in electrospinning technology (such as multifluid electrospinning to generate complex nanostructures and Janus structure-based advanced nanofibers) have been used in biomedical applications [40,41].

In this study, we used electrospinning technology to synthesize resveratrol-loaded nanofibers containing different ratios of polyvinylpyrrolidone (PVP) and hydroxypropyl-β-cyclodextrin (HPBCD). We determined whether resveratrol nanofibers can effectively improve the aqueous solubility of resveratrol, analyzed their surface morphology and physicochemical properties, determined their skin penetration ability, and investigated their antioxidant and anti-inflammatory activities.

## 2. Materials and Methods 

### 2.1. Materials

Resveratrol was obtained from Acetar Bio-Tech Inc. (Xi’an, China). The purity of resveratrol was greater than 97% as determined by high-performance liquid chromatography (HPLC). PVP (average molecular weight 1,300,000 g/mol) was acquired from Sigma-Aldrich (St. Louis, MO, USA). HPBCD was purchased from Zibo Qianhui Biological Technology Co., Ltd (Shandong, China). PM (1649b) was purchased from the National Institute of Standards and Technology (Gaithersburg, MD, USA).

### 2.2. Electrospinning Procedure

The electrospinning procedure was performed using FES-COS Electro-spinning equipment (Falco Tech Enterprise Co., Taipei, Taiwan). The amount of resveratrol, PVP and HPBCD used to prepare different ratio formulations of resveratrol nanofibers during the electrospinning procedure is presented in Table 1. Firstly, 0.05 g of resveratrol was dissolved in 10 ml of ethanol, and mixed evenly using a magnetic stirrer. HPBCD was then added, and mixed with a magnetic stirrer until it fully dissolved, to ensure that resveratrol had been completely encapsulated by HPBCD. Subsequently, PVP was added, and mixed with a magnetic stirrer till it fully dissolved. The evenly mixed solution was transferred into a syringe with micropump attached. After the electrospinning process, the nanofibers were collected by an aluminum foil. The newly synthesized nanofibers were placed in a sealed plastic bag and stored in a moisture-proof container.

### 2.3. High-Performance Liquid Chromatography

High-performance liquid chromatography (HPLC) was performed using a pump with an ultraviolet detector system (Hitachi, Tokyo, Japan). The Mightysil RP-18 GP column (internal diameter of 250 mm × 4.6 mm, particle size 5 μm) was used for HPLC (Kanto Corporation, Portland, OR, USA). The mobile phase was composed of acetonitrile : KH_2_PO_4_ (ratio 35:65, pH 2.8). The flow rate was 1 ml/min. The eluted resveratrol was detected by an ultraviolet detector (wavelength 290 nm). The absorption peak for resveratrol appeared at 6.57 min.

To obtain the standard calibration curve for HPLC, 1 mg of resveratrol was dissolved in 1 ml methanol, and filtered through a 0.45-μm membrane. The resveratrol solution was serially diluted to 100, 50, 10, 5, 1, 0.5, 0.1, 0.05, and 0.01 μg/mL, and analyzed by HPLC. A standard curve for resveratrol was then constructed.

### 2.4. Determination of Entrapment Efficiency

Firstly, 10 mg of different ratio formulations of resveratrol nanofibers was dissolved in 1 ml methanol, filtered through a 0.45-μm membrane (Pall Corporation, Port Washington, NY, USA), and diluted 25-fold. The diluted solutions were analyzed by HPLC, and the standard curve was used to calculate the resveratrol amount. The following equation was used to calculate entrapment efficiency [42]: 

Resveratrol entrapment efficiency (%) = (Amount of drug in nanofiber / Amount of initially used drug) × 100%.

### 2.5. Determination of Aqueous Solubility

Measurement of water solubility was performed at 25 °C temperature and 50 ± 5% humidity. Pure resveratrol (1 mg) and different ratio formulations of resveratrol nanofibers (containing equivalent of 1 mg resveratrol) were dissolved in 1 ml deionized water, shaken for 1 h (Vortex-Gene 2, Scientific Industries, Bohemia, NY, USA), filtered through a 0.45-μm membrane (Pall Corporation), and diluted 10-fold. The diluted solutions were analyzed by HPLC, and the standard curve was employed to determine the resveratrol amount.

### 2.6. Scanning Electron Microscopy

The surface morphology of the nanofiber formulations was visualized using scanning electron microscopy. The nanofibers were sputter coated with gold–palladium and then visualized by a scanning electron microscope (Hitachi S4700, Hitachi, Tokyo, Japan). 

### 2.7. X-Ray Diffractometry

X-ray diffractometry (Siemens D5000, Siemens, Munich, Germany) was used to determine the crystalline versus amorphous structure of pure resveratrol and resveratrol nanofiber formulations. The analysis was conducted using nickel-filtered Cu-Kα radiation, using a voltage of 40 kV and current of 25 mA. The range of the angles scanned was from 5° to 50°.

### 2.8. Fourier Transform Infrared (FTIR) Spectroscopy

The samples (pure resveratrol, PVP, HPBCD and different ratio formulations of resveratrol nanofibers) were mixed with potassium bromide using a mortar, and compressed to form thin sheets. The samples were then analyzed by a FTIR spectrophotometer (Perkin-Elmer Inc., Waltham, MA, USA).

### 2.9. DPPH Free Radical Scavenging Activity

2,2-diphenyl-1-picrylhydrazyl (DPPH) is a lipophilic free radical, and is soluble in ethanol. It has a purple color, and absorbs light strongly at a wavelength of 517 nm. When DPPH undergoes a redox reaction with an antioxidant, the color of DPPH turns to yellow, and the absorption at 517 nm is decreased [43].

The experimental groups were pure resveratrol in water, pure resveratrol in methanol, resveratrol nanofibers, and vitamin C (as a reference antioxidant). The samples were filtered and serially diluted. To perform the DPPH free radical scavenging assay, 100 μL of different concentrations of the test samples was mixed with 100 μL DPPH (200 μM) in a 96-well plate, and incubated in the dark for 30 min. The absorption was then measured using a spectrometer at a wavelength of 517 nm.

### 2.10. Reducing Power Assay 

The production of Prussian blue (Fe_4_[Fe(CN)_6_]_3_) is used to assess reducing power. For this assay, the Fe^3+^ in [K_3_Fe(CN)_6_] is first reduced by the test sample to Fe^2+^ in [K_4_Fe(CN)_6_], and then the solution is reacted with Fe^3+^ in FeCl_3_ to form Prussian blue. The absorption at 700 nm is measured to determine the concentration of Prussian Blue [44].

The reducing power of pure resveratrol and resveratrol nanofibers was evaluated against vitamin C as a reference antioxidant. To perform this assay, 250 μL of the test sample, 250 μL of phosphate buffer (0.2 M), and 250 μL of K_3_Fe(CN)_6_ (1%) were mixed in a 1.5-ml microtube at 50 °C for 20 min, and placed in a cool water bath for 2 min. Then, 250 μL of trichloroacetic acid solution (10%) was added and the solution was centrifuged at 3000 rpm at 4 °C for 10 min. After centrifugation, 100 μL of supernatant was mixed with 140 μL deionized water and 10 μL FeCl_3_·6H_2_O (0.1%) in a 96-well plate and incubated in the dark for 10 min. The absorption was then measured using a spectrometer at a wavelength of 700 nm.

### 2.11. ABTS Cation Free Radical Scavenging Activity

When ABTS (2,2’-azino-bis (3-ethylbenzothiazoline-6-sulfonic acid)) is oxidized, it forms a blue-green colored cation free radical ABTS+. When an antioxidant test sample is added, ABTS+ is reduced back to ABTS, and the solution becomes colorless [45]. 

The ABTS radical scavenging activity of pure resveratrol and resveratrol nanofibers was evaluated against vitamin C as a reference antioxidant. To perform this assay, ABTS (7 mM) was mixed with K_2_S_2_O_8_ (2.45 mM) in a 1:1 ratio, and incubated for 12~16 h. The ABTS solution (170 μL) was then mixed with the test sample (30 μL) in a 96-well plate. The absorption was measured using a spectrometer at a wavelength of 734 nm.

### 2.12. Ex Vivo Skin Penetration

This experiment was performed according to the European Cosmetic Toiletry and Perfumery Association (COLIPA) guideline [46]. The Franz diffusion cell consists of two compartments, the upper donor chamber and the lower receptor chamber. Pig skin from the flank region was cut into 2 cm × 2 cm pieces and placed between the two chambers, with the stratum corneum facing upwards. The Franz diffusion cell was maintained at 32 °C and stirred at 600 rpm throughout the experiment. To measure skin penetration, 200 μL of each sample (n = 6) was added to the donor chamber. After different amounts of time (1, 2, 4, and 8 h), the pig skin was removed from the Franz diffusion cell. The stratum corneum was obtained by tape stripping. To obtain the epidermal and dermal layers, the skin sample was heated to 85 °C with a heat pad, and the epidermis and dermis were separated with a scalpel. The samples were immersed in methanol and sonicated for 1 h to extract resveratrol. The content of resveratrol in each sample was determined by the HPLC method.

### 2.13. Western Blotting 

HaCaT cells were cultured in 6-well plates, and treated with pure resveratrol or resveratrol nanofibers. Cells were then harvested, treated with lysis buffer, and proteins were extracted. The proteins were separated by sodium dodecylsulfate–polyacrylamide gel electrophoresis (SDS-PAGE), and then blotted onto polyvinylidene difluoride (PVDF) membranes. The membranes were incubated with antibodies against cyclooxygenase-2 (COX-2) (Santa Cruz Biotechnology, Dallas, TX, USA) and matrix metalloproteinase-9 (MMP-9) (Cell Signaling Technology, Danvers, MA, USA) overnight at 4 °C. Secondary antibodies were then added for 1 h at room temperature, and the immunoreactive bands were visualized with the enhanced chemiluminescence (ECL) system. Antibodies against GAPDH and α-tubulin were used as internal controls. 

### 2.14. Statistical Analysis

Data are shown as mean ± standard error of the mean (SEM). Comparisons between multiple groups were performed using analysis of variance (ANOVA) with post-hoc Tukey’s test. P value < 0.05 was considered to be statistically significant.

## 3. Results

### 3.1. Entrapment Efficiency and Aqueous Solubility

Different ratio formulations of resveratrol nanofibers were subjected to HPLC analysis, and the amount of resveratrol was determined from the standard curve. It was found that ratios of 1:4:20, 1:4:40, 1:8:40 and 1:16:40 (Res:PVP:HPBCD) all had greater than 90% entrapment efficiency (Table 2). In particular, the ratio of 1:4:20 produced the greatest entrapment efficiency. In addition, the different ratio formulations of resveratrol nanofibers all had aqueous solubility greater than 800 μg/mL, indicating that the nanofiber formulations increased the solubility of resveratrol by more than 20,000-fold. 

### 3.2. Surface Morphology of Resveratrol and Resveratrol Nanofibers

**Figure 2:**
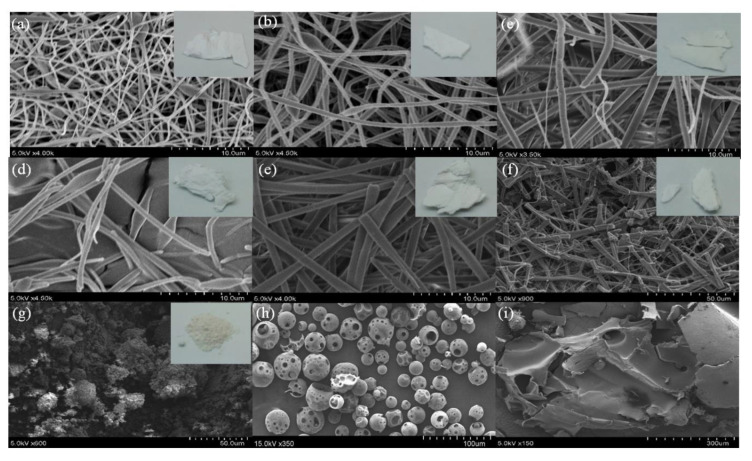
Surface morphology of resveratrol, HPBCD, PVP, and different ratio formulations (Res:PVP:HPBCD) of resveratrol nanofibers, as determined by scanning electron microscopy. (**a**) 1:4:20, (**b**) 1:8:20, (**c**) 1:16:20, (**d**) 1:4:40, (**e**) 1:8:40, (**f**) 1:16:40, (**g**) pure resveratrol, (**h**) pure HPBCD, (**i**) pure PVP.

Scanning electron microscopy of resveratrol nanofibers showed that a Res:PVP:HPBCD ratio of 1:4:20 produced the thinnest nanofibers (437.96 ± 41.92 nm) (Table 3, Figure 2). As the proportions of PVP and HPBCD were increased, the diameter of the nanofibers also increased. In particular, when the ratio reached 1:16:40, the structure became fragmented and continuous nanofibers could not be formed. On the other hand, scanning electron microscopy of pure resveratrol showed a clustered particle-like appearance, pure HBPCD exhibited globular structures with cavities, while pure PVP showed plate-like structures.

### 3.3. Crystalline versus Amorphous Structure of Resveratrol and Resveratrol Nanofibers

**Figure 3:**
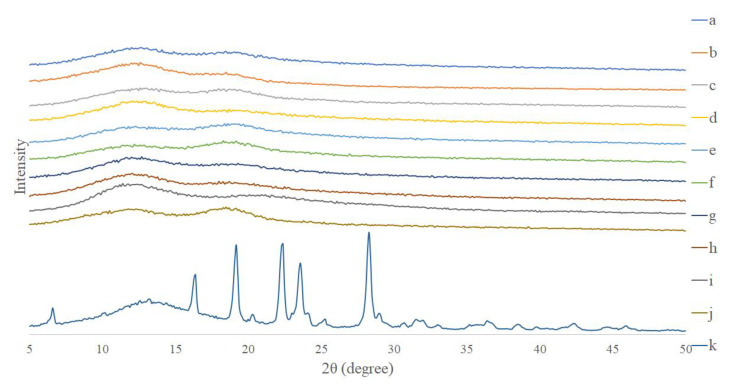
X-ray diffractometry patterns of resveratrol, PVP, HPBCD, and different ratio formulations (Res : PVP : HPBCD) of resveratrol nanofibers. (**a**) 1:16:40, (**b**) 1:8:40, (**c**) 1:4:40, (**d**) 1:16:20, (**e**) 1:8:20, (**f**) 1:4:20, (**g**) PVP:HPBCD = 1:5 (blank nanofiber), (h) physical mixture of PVP and HPBCD, (i) pure PVP, (j) pure HPBCD, (k) pure resveratrol.

The X-ray diffractometry results showed that pure resveratrol exhibited multiple diffraction peaks at 5°~30°, indicating that resveratrol had a crystalline structure (Figure 3). On the other hand, the X-ray diffractometry patterns of pure PVP and HPBCD did not display definite diffraction peaks. Following nanofiber formation from the electrospinning procedure (resveratrol nanofibers), the diffraction peaks for resveratrol disappeared and the curves resembled that of PVP and HPBCD. These results showed that following nanofiber formation, resveratrol had been converted from a crystalline to an amorphous structure, indicating that resveratrol had been encapsulated by the nanocarriers.

### 3.4. Intermolecular Chemical Bond Formation Between Resveratrol, HPBCD and PVP

**Figure 4:**
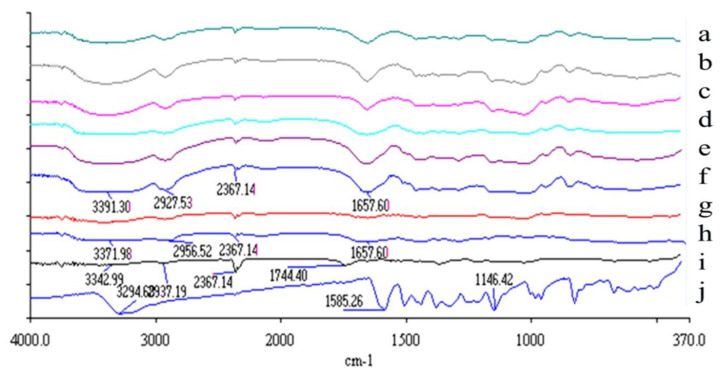
Fourier transform infrared (FTIR) spectra for resveratrol, PVP, HPBCD, and different ratio formulations (Res:PVP:HPBCD) of resveratrol nanofibers. (**a**) 1:16:40, (**b**) 1:8:40, (**c**) 1:4:40, (**d**) 1:16:20, (**e**) 1:8:20, (**f**) 1:4:20, (**g**) physical mixture of PVP and HPBCD, (**h**) pure PVP, (**i**) pure HPBCD, (**j**) pure resveratrol.

The intermolecular interactions between resveratrol, HPBCD and PVP were analyzed using FTIR. The FTIR spectra showed that pure resveratrol had an absorption band at 3200~3500 cm^-1^ (corresponding to OH functional groups) (Figure 4). PVP showed an absorption band at 1600~1700 cm^-1^ (corresponding to C=O functional group). When resveratrol was complexed with PVP and HPBCD to form resveratrol nanofibers, the absorption band at 3200~3500 cm^-1^ was broadened (indicating formation of new hydrogen bonds with nanocarriers), and there was an absorption band at 1600~1700 cm^-1^ (corresponding to C=O functional group of PVP). These results indicate that resveratrol had formed new intermolecular bonds, and had been encapsulated by its nanocarriers. 

### 3.5. DPPH Free Radical Scavenging Activity

**Figure 5:**
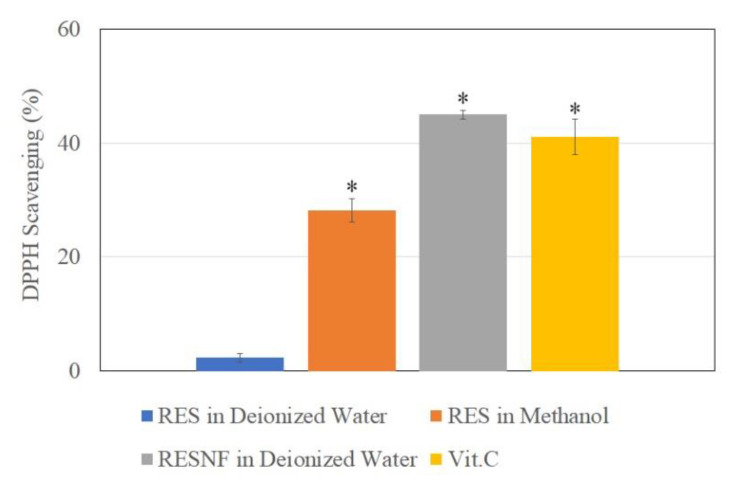
DPPH free radical scavenging activity of resveratrol in deionized water, resveratrol in methanol, resveratrol nanofibers (RESNF) in deionized water, and vitamin C at 10 μg/mL concentration. Results are shown as mean ± SEM of three independent experiments. * represents P < 0.05 when compared to resveratrol in deionized water.

The in vitro antioxidant assays were performed using a ratio of 1:4:20 for resveratrol nanofibers. The results from the DPPH assay showed that the free radical scavenging activity of resveratrol nanofibers was greater than that of pure resveratrol in methanol, and equivalent to that of vitamin C (Figure 5). This indicates that the antioxidant activity of resveratrol was improved following nanofiber formation. The pure resveratrol in water formulation showed low DPPH free radical scavenging activity, likely due to the poor water solubility of resveratrol.

### 3.6. Reducing Power

**Figure 6:**
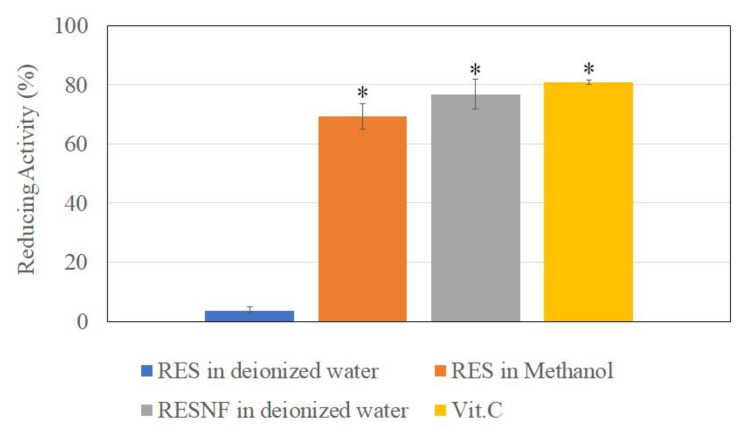
Reducing power of resveratrol in deionized water, resveratrol in methanol, resveratrol nanofibers (RESNF) in deionized water, and vitamin C at 20 μg/mL concentration. Results are shown as mean ± SEM of three independent experiments. * represents P < 0.05 when compared to resveratrol in deionized water.

The results from the reducing power assay (production of Prussian blue) showed that the reducing power of pure resveratrol in methanol and resveratrol nanofibers was similar and equivalent to that of vitamin C (Figure 6). Therefore, resveratrol retained its reducing power following nanofiber formation. On the other hand, pure resveratrol in water showed low reducing power, likely due to the poor aqueous solubility of resveratrol.

### 3.7. ABTS Cation Free Radical Scavenging Activity

**Figure 7:**
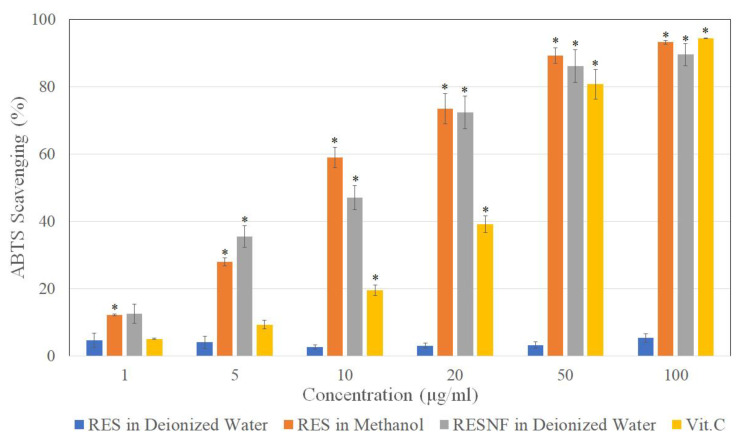
ABTS free radical scavenging activity of resveratrol in deionized water, resveratrol in methanol, resveratrol nanofibers (RESNF) in deionized water, and vitamin C at various concentrations. Results are shown as mean ± SEM of three independent experiments. * represents P < 0.05 when compared to resveratrol in deionized water.

The ABTS free radical scavenging activity of pure resveratrol in methanol and resveratrol nanofibers was similar and equivalent to that of vitamin C (Figure 7). This indicates that resveratrol retained its ABTS free radical scavenging activity following nanofiber formation. On the other hand, pure resveratrol in water showed low ABTS scavenging activity, likely due to the poor solubility of resveratrol in water.

### 3.8. Ex Vivo Skin Penetration

**Figure 8:**
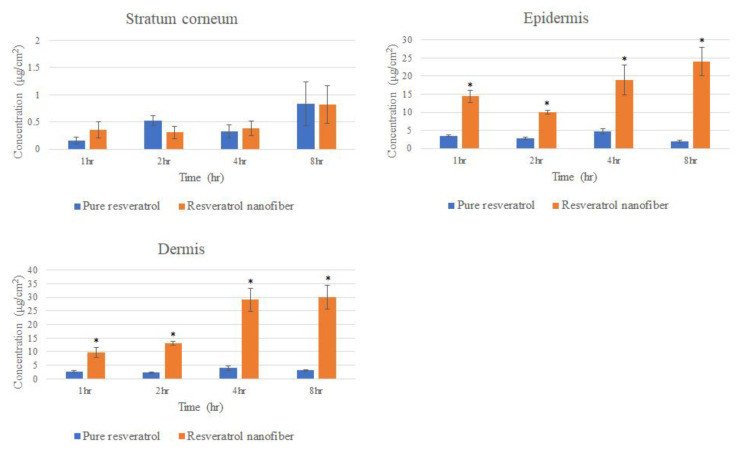
Effects of pure resveratrol and resveratrol nanofibers on skin penetration through pig skin. The amount of drug in the stratum corneum, epidermis and dermis at various time points were determined. Results are shown as mean ± SEM (n = 6). * represents P < 0.05 when compared to pure resveratrol.

We next investigated whether the skin penetration ability of resveratrol is increased after nanofiber formation. Pig skin was exposed to pure resveratrol or resveratrol nanofibers, and different layers of the skin (stratum corneum, epidermis, dermis) were collected at 1, 2 and 4 and 8 h to determine drug concentration. The results demonstrated that resveratrol in the nanofiber formulation showed greater penetration into the epidermis and dermis compared with pure resveratrol, and this occurred in a time-dependent manner (Figure 8). Therefore, nanofiber formulations of resveratrol may increase the skin penetration of resveratrol.

### 3.9. Effects of Resveratrol Nanofibers on Particulate Matter (PM)-Induced Inflammatory Proteins Expression in HaCaT Keratinocytes 

**Figure 9:**
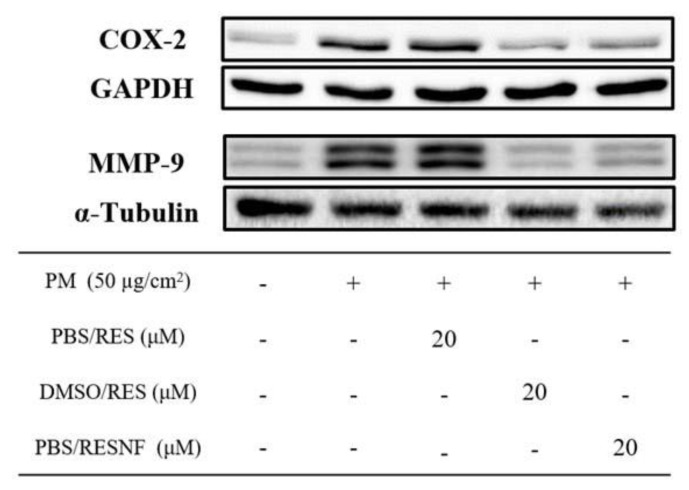
Western blots demonstrating the effects of pure resveratrol in PBS (PBS/RES), pure resveratrol in DMSO (DMSO/RES) and resveratrol nanofibers in PBS (PBS/RESNF) on particulate matter (PM)-induced protein expression of COX-2 (24 h) and MMP-9 (24 h) in HaCaT keratinocytes. Blots are representative of three independent experiments.

Western blotting showed that treatment of HaCaT keratinocytes with PM (50 μg/cm^2^) led to increased protein expression of inflammatory proteins COX-2 and MMP-9 (Figure 9). Treatment of HaCaT keratinocytes with pure resveratrol in PBS (20 μM) had no significant effect on the expression levels of these proteins, which may be due to the poor aqueous solubility of resveratrol. On the other hand, treatment of HaCaT cells with pure resveratrol in DMSO (20 μM) and resveratrol nanofibers (20 μM) suppressed PM-induced expression of COX-2 and MMP-9. Therefore, resveratrol retained its anti-inflammatory effects following nanofiber formation.

## 4. Discussion

Nanofibers generated from electrospinning technology have been increasingly used as drug delivery systems [37,38,47]. In this study, we synthesized nanofibers of PVP and cyclodextrin (HPBCD) loaded with resveratrol, in order to improve the solubility and physicochemical properties of the drug. The use of nanofibers as drug carriers present several advantages, including high loading capacity and encapsulation efficiency, and a high surface area to volume ratio leading to increased dissolution rate. PVP is chosen as a nanocarrier in this study since it is characterized by high water solubility, rapid dissolution and low toxicity [48,49,50]. However, PVP is highly hygroscopic and its nanofibers are unstable in atmospheric conditions [50]. Blending HBDCD with PVP can reduce the hygroscopicity of PVP and improve the stability of the nanofibers [51]. Previously, nanofiber formulations combining active compounds with PVP and HPBCD have been synthesized using electrospinning technology [50,51,52,53]. 

In this study, we found that resveratrol can be complexed with HPBCD/PVP and subjected to the electrospinning procedure to form stable nanofibers. The electrospinning process achieved good entrapment efficiency, and the water solubility of resveratrol nanofibers was greatly increased compared to the pure compound. HPBCD is a cyclodextrin with high water solubility, and is characterized by a lipophilic inner cavity and hydrophilic outer surface [54]. The inclusion of resveratrol into the cavity of HPBCD to form a cyclodextrin inclusion complex may explain the greatly increased aqueous solubility of resveratrol [33]. Moreover, the encapsulation of resveratrol by the nanocarrier PVP may lead to improved water solubility. For future work, it will be important to test the in vitro release profile of resveratrol from nanofibers, in order to gain further insight into the pharmaceutical properties of this drug formulation.

Scanning electron microscopy showed that resveratrol nanofibers with low proportions of PVP and HPBCD (for example, Res:PVP:HPBCD ratio of 1:4:20) produced the thinnest nanofibers. On the other hand, resveratrol nanofibers with high proportions of PVP and HPBCD (in particular 1:16:40) produced thicker fibers which became fragmented. A possible explanation is that when the proportions of PVP and HPBCD increase, the viscosity of the nanofibers also increases [50,55], which may lead to the formation of thicker fibers. In addition, the formation of intermolecular bonds between resveratrol and HPBCD/PVP may stabilize the nanofiber structure, enabling the formation of thin continuous nanofibers.

The improvement in water solubility of resveratrol may be a result of changes in physicochemical properties. An analysis of the physicochemical properties by X-ray diffractometry demonstrated that following nanofiber formation, resveratrol was converted from a crystalline to amorphous structure. In addition, FTIR showed that new intermolecular bonds (including hydrogen bonds) were formed between resveratrol and HPBCD/PVP. These results indicate that resveratrol had been encapsulated by the nanocarriers, and the formation of nanofibers improved the physicochemical properties of resveratrol. Previous reports have also demonstrated that when a drug is loaded into PVP/HPBCD nanofibers, it is converted from crystalline to amorphous form, and new intermolecular bonds are formed between the drug and nanocarriers [50,51].

Moreover, we demonstrated that resveratrol nanofibers possess good DPPH free radical scavenging activity, reducing power, and ABTS radical scavenging activity, indicating that resveratrol may show improved antioxidant activity following nanofiber formation [3]. Furthermore, the antioxidant activities of resveratrol nanofibers are equivalent to that of vitamin C, which is known as a strong natural antioxidant [56]. Therefore, nanofiber formulations of resveratrol not only show greatly improved water solubility, but exhibit good antioxidant activity as well. Our results indicate that resveratrol-loaded PVP/HPBCD nanofibers may have potential clinical applications as an antioxidant formulation in the future. 

In order to exert biological effects on the skin, cutaneous drug formulations need to penetrate the different layers of skin. The stratum corneum has been shown to be a rate-limiting factor for the skin penetration of drugs [57]. In this study, pig skin was used to investigate skin penetration, since it exhibits cutaneous barrier function similar to human skin [58]. Our results demonstrated that, compared to pure resveratrol, resveratrol in the nanofiber formulation showed greater penetration through the stratum corneum and into the epidermal and dermal layers. Therefore, resveratrol nanofibers may be a suitable cutaneous drug formulation.

Previously, it has been shown that particulate matter (PM) in air pollution may exert inflammatory effects on the skin [59,60,61,62]. Therefore, we used PM-induced HaCaT keratinocyte inflammation as an in vitro model to investigate the potential anti-inflammatory effects of resveratrol nanofibers. Proteins which have been shown to play important roles in inflammation include COX-2 [63] and MMP-9 [64,65,66]. Our results showed that resveratrol nanofibers suppressed particulate matter (PM)-induced expression of inflammatory proteins COX-2 and MMP-9 in keratinocytes. Therefore, resveratrol nanofibers may be a potential anti-inflammatory drug formulation for skin.

Based on the results of this study, we propose that resveratrol nanofibers may be a suitable cosmetic or drug formulation for topical skin application. The nanofibers may be applied to the skin as a thin film with local actions on the skin, for example as a facial mask product. The high surface area to volume ratio of the nanofiber facial mask may facilitate drug delivery to facial skin, leading to antioxidant and anti-inflammatory actions. Previously, facial masks formed from electrospun fiber mats have been developed to exert an anti-wrinkle effect on facial skin [67]. Since oxidative stress and chronic inflammation are known to play important roles in skin aging [68], resveratrol nanofibers may be a potential anti-aging cosmetic formulation.

In conclusion, resveratrol-loaded nanofibers can effectively improve the aqueous solubility and physicochemical properties of resveratrol, show enhanced skin penetration, and exhibit antioxidant and anti-inflammatory properties. Therefore, resveratrol nanofibers may have potential applications as an antioxidant and anti-inflammatory formulation for topical skin application in the future.

## Figures and Tables

**Table 1:** Amounts of resveratrol, PVP and HPBCD in different ratio formulations of resveratrol nanofibers.

Ratio (Res:PVP:HPBCD, W/W/W)	Resveratrol (g)	PVP (g)	HPBCD (g)
1:4:20	0.05	0.2	1
1:8:20	0.05	0.4	1
1:16:20	0.05	0.8	1
1:4:40	0.05	0.2	2
1:8:40	0.05	0.4	2
1:16:40	0.05	0.8	2

**Table 2:** Entrapment efficiency and water solubility of different ratio formulations of resveratrol nanofibers determined by HPLC analysis.

Ratio (Res:PVP:HPBCD, W/W/W)	Entrapment Efficiency (%)	Solubility (μg/mL)
1:04:20	98.27 ± 4.59	894.90 ± 22.02
1:08:20	84.54 ± 5.98	875.68 ± 23.83
1:16:20	79.00 ± 8.21	855.50 ± 35.20
1:04:40	97.31 ± 5.24	860.34 ± 22.30
1:08:40	96.01 ± 5.60	900.63 ± 31.47
1:16:40	90.16 ± 7.67	896.43 ± 25.46
Pure resveratrol	-	0.041 ± 0.001

Results are shown as mean ± SEM of three independent experiments.

**Table 3:** Nanofiber diameter for different ratio formulations of resveratrol nanofibers determined by scanning electron microscopy.

Ratio (Res:PVP:HPBCD, W/W/W)	Nanofiber Diameter (nm)
1:04:20	437.96 ± 41.92
1:08:20	578.66 ± 40.11
1:16:20	906.51 ± 61.85
1:04:40	835.78 ± 79.57
1:08:40	1259.59 ± 44.29
1:16:40	2639.59 ± 157.00

Results are shown as mean ± SEM.
